# Monkeypox (Mpox), a Resurging Global Public Health Concern: An Updated Outlook Through 2025

**DOI:** 10.3390/cimb48040340

**Published:** 2026-03-24

**Authors:** Dewan Zubaer Islam, Fahmida Sultana Tamanna, Mohtasim Fuad, Mst. Sanzida Akter Shanta, Akhi Khanom, Md. Mehedi Hasan, Md. Shiful Islam Sujan, Shahad Saif Khandker, Md Shahin Reza, Salma Akter, Md. Firoz Ahmed, Nafisa Azmuda, Nihad Adnan, Abu Ali Ibn Sina

**Affiliations:** 1 Department of Microbiology, Jahangirnagar University, Dhaka 1342, Bangladesh; dewanzubaerislam@gmail.com (D.Z.I.); fahmidasultana2017@gmail.com (F.S.T.); akhikhanom1999@gmail.com (A.K.); mehediju13@gmail.com (M.M.H.); sujanbd1384@gmail.com (M.S.I.S.); salma_akter025@juniv.edu (S.A.); firoz@juniv.edu (M.F.A.); azmuda@juniv.edu (N.A.); 2 Department of Zoology, Jahangirnagar University, Dhaka 1342, Bangladesh; mohtasimtanim@gmail.com (M.F.); sanzidashanta1904@gmail.com (M.S.A.S.); 3 Department of Biochemistry and Molecular Biology, Gono Bishwabidyalay, Dhaka 1344, Bangladesh; shahadsaifkhandker@gmail.com; 4 Department of Chemistry, Tennessee State University, Nashville, TN 37209, USA; shahinreza.ju@gmail.com; 5 School of Biotechnology and Biomolecular Sciences, The University of New South Wales (UNSW), Sydney, NSW 2300, Australia

**Keywords:** epidemiology, transmission, diagnosis, vaccine, monkeypox, mpox, evolution, pathogenesis, treatment, prevention

## Abstract

Monkeypox (Mpox) disease, caused by the Monkeypox virus (Mpox virus), emerged as a significant global health threat during the 2022 outbreak, prompting the World Health Organisation (WHO) to declare it a Public Health Emergency of International Concern (PHEIC). Rapid evolution through genomic modifications enhanced its outbreak potential. Zoonotic transmission occurs through close contact with infected rodents or primates; human-to-human transmission occurs via close contact or homosexual intercourse. The virus disseminates via the lymphatic system, causing symptoms ranging from mild skin lesions to severe multi-system complications or even death. Diagnosis incorporates clinical symptoms as well as advanced molecular and immunological methods. Currently, no specific antiviral medications or vaccines are available for Mpox, necessitating reliance on conventional therapeutic supports and treatments developed for smallpox. Raising awareness, promoting protective practices, implementing surveillance, enabling rapid diagnosis, ensuring timely treatment, and promoting mass vaccination are crucial to curb Mpox transmission. This narrative review provides a comprehensive overview of the current knowledge on epidemiology, evolution, transmission, pathogenesis, clinical signs, diagnosis, treatment, vaccination, and prevention strategies for Mpox.

## 1. Introduction

The Monkeypox virus (Mpox virus), belonging to the *Orthopoxvirus* genus and *Poxviridae* family, causes a zoonotic illness that can be transmitted from animals to humans and from person to person. This disease is characterised by the appearance of a pustular rash [1] and exhibits signs similar to those of smallpox [2]. The virus was first identified in Denmark in 1958, with the first human case reported in the Democratic Republic of Congo in 1970. Initially confined to remote regions of Africa, the Mpox virus first appeared in the United States during an outbreak in 2003. Concerns have emerged regarding evolving epidemiological trends and global health threats, as Mpox cases increased significantly from 2018 to 2024 [3,4]. According to a report published by the World Health Organisation (WHO) on 29 April 2025, 137,892 Mpox cases have been confirmed globally between 1 January 2022, and 31 March 2025, with 317 deaths across 132 countries [4]. Notably, on 14 August 2024, the WHO classified the Mpox virus, a zoonotic pathogen, as a public health emergency of international concern (PHEIC) [5].

The distribution of Mpox cases has been predominantly observed in Africa from 2022 to 2025, with the Democratic Republic of the Congo (DRC) being significantly impacted by the Ia and Ib clades of the virus. Conversely, Clade IIb has been primarily concentrated in Sierra Leone. The WHO assessed the public health risk as high for clade Ib and moderate for other clades, underscoring the need for continued surveillance and response measures [6,7]. Mpox infection is heightened in rural, wooded areas of Central and Western Africa, particularly among individuals who handle bush meat, caregivers of infected persons, unvaccinated individuals, and men who hunt and interact with wild animals [8]. Infected individuals typically display distinct symptoms, which include fever, multiple papular lesions, vesiculopustular lesions, and ulcerative lesions on the body and face, accompanied by lymphadenopathy [7,9,10,11,12,13]. Upon entry, the Mpox virus replicates primarily within immune cells and disseminates to regional lymph nodes. After lymphatic spread, the virus infiltrates various tissues throughout the body [14].

Symptoms typically onset between 7 and 14 days post-infection, with prodromal signs including headaches, fever, and chills that may appear 1 to 2 days before the onset of a facial rash. In more severe cases, the infection can lead to critical complications such as pneumonia, encephalitis, or septicemia, which may result in fatal outcomes. The infected individuals remain contagious throughout this period [15,16]. The clinical features of Mpox are similar to those of smallpox; however, certain symptoms help distinguish the two diseases. Notably, the presence of lymphadenopathy, or swollen lymph nodes, is a defining characteristic of Mpox that is absent in smallpox [17]. Mpox infections are usually self-limiting, and milder cases can be managed with supportive care [18]. People with weakened immune systems, aged less than 8 years, pregnant women, or those with an immunocompromised state or damaged skin are at greater risk of severe Mpox. Currently, no specific treatment exists for Mpox. Various antiviral medications and vaccines originally designed and approved for smallpox or other orthopoxviruses have been used in the treatment of Mpox [18].

The potential for asymptomatic transmission of the Mpox virus is a major concern, especially since the routine smallpox vaccination program ended in 1980, which may have altered the virus’s structure. Changes in human behaviour and movement contributed to the 2022 outbreaks, yet limited research exists on their epidemiology, clinical features, and transmission, unlike previous rare outbreaks [2,7,19]. The WHO (2022) noted cases without travel or contact from endemic areas, indicating unknown routes and a pandemic risk [20]. Additionally, a thorough investigation has shown that the fatality rate for Mpox cases ranges from 1% to 11% [21].

Currently, there are few up-to-date, Mpox-specific vaccines or treatments available, highlighting the need for immunity-boosting vaccines and better professional knowledge, as confidence in diagnosis and management remains low [22,23]. Increased awareness allows for quick diagnosis, contact tracing, isolation, and outbreak control, even though diverse symptoms can delay testing and increase patient anxiety [24]. This highlights the urgent need for educational initiatives and training to prevent and effectively manage the current outbreak [18]. The surge in cases has prompted a greater focus on investigating the clinical features and transmission of the outbreak to attain a clearer understanding of the situation.

This narrative review aims to present an in-depth overview of the genomic characteristics, evolution, and current epidemiological landscape of the Mpox outbreak, and to explore various signs and symptoms associated with the infection, as well as the modes of transmission and the underlying mechanisms of its pathogenesis. Additionally, the review will discuss the major diagnostic methods available, covering both the conventional approaches and innovative detection techniques. A comprehensive analysis of treatment options will be included, emphasising both traditional approaches and newer antiviral medications. Finally, the review will address preventive strategies, including vaccines from diverse platforms, designed to combat the spread of Mpox.

## 2. Methodology

**Figure 1:**
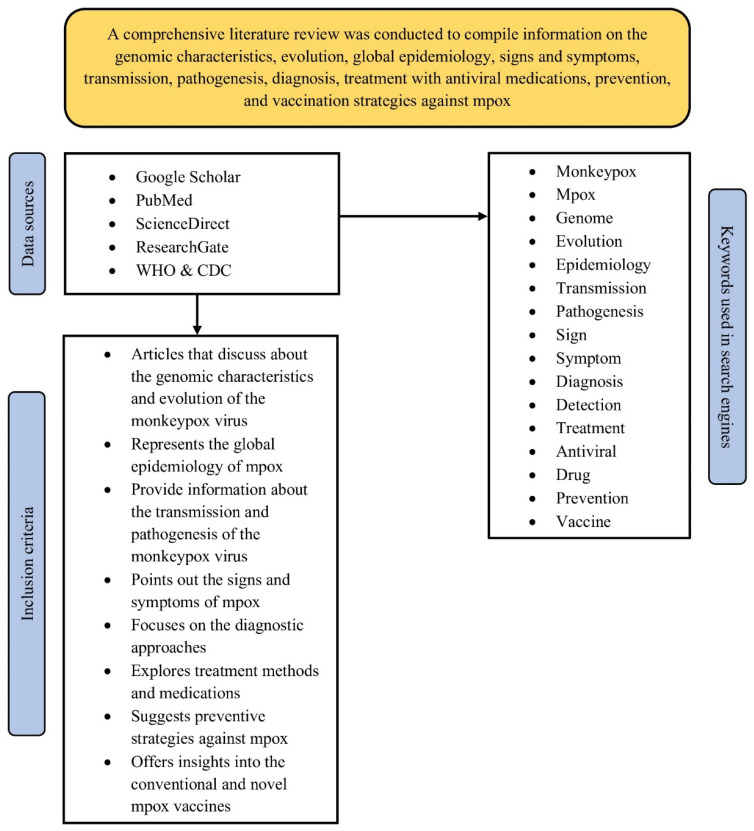
Schematic flowchart presenting the literature search strategy of the review.

To gather relevant data for this review, articles were retrieved from three major databases: Google Scholar, PubMed, and ScienceDirect. Specific search keywords such as “genome”, “evolution”, “epidemiology,” “signs,” “symptoms,” “complication”, “transmission,” “pathogenesis,” “diagnosis,” “detection,” “treatment,” “antiviral,” “drug,” “vaccine,” and “prevention” were combined with “Mpox” and “mpox” using appropriate Boolean operators, i.e., AND and OR. Additionally, “Title and abstract” and “Title, abstract, or author-specified keywords” filters were employed in the advanced search options on PubMed and ScienceDirect, respectively, while the term “allintitle” was used prior to the specific search keywords during searches in Google Scholar. Additional filters, such as “Full text,” were applied in PubMed and ScienceDirect, respectively. Relevant data were also gathered from articles available on ResearchGate, as well as from various recommendations, guidelines, and reports published by the World Health Organisation (WHO) and the Centres for Disease Control and Prevention (CDC) (Figure 1).

## 3. Review of Literature

### 3.1. Evolution of Mpox Virus and Global Distribution

Various genomic modifications, including variations in mutation types and rates, genetic recombination, and the loss of specific genes, have contributed to Mpox virus evolution and changes in viral host specificity. One significant driver of Mpox virus evolution is the increased frequency of G>A/C>T substitutions, which can be attributed to the action of the host apolipoprotein B mRNA editing enzyme, catalytic subunit 3 (APOBEC3) cytidine deaminase. This activity generates mutations that enhance adaptability while potentially reducing viral fitness. Notably, APOBEC3 is responsible for 87% of the alterations observed in strains from the 2022 outbreak, particularly in genes that regulate host interactions. Furthermore, evidence suggests that undetected transmission has occurred since 2016, and this mutational bias underscores the virus’s prolonged circulation within human populations [25,26].

**Figure 2:**
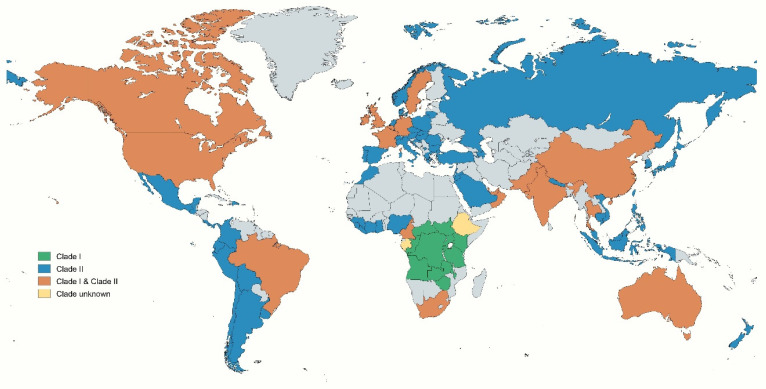
The world map illustrates the global distribution of different clades of the Mpox virus. Distinct clades are represented by individual colours, while grey-shaded regions indicate areas with no reported cases.

Phylogenomic analysis reveals that clade I of the Mpox virus, particularly in South Kivu, exhibits greater genetic diversity and is evolving towards enhanced human-to-human transmission through APOBEC3-type mutations. These mutations not only promote viral replication but also enable the virus to evade the host’s immune system. In contrast, clade II b, linked to the 2022–2023 outbreak, demonstrates more stable genetic variation and a slower evolutionary rate [27]. The Mpox virus is classified into two clades: clade I, known as the Central African/Congo Basin clade, and clade II, the West African clade, which is further divided into IIa and IIb. Clade IIb exhibits a lower mortality rate of less than 1% but demonstrates more efficient dissemination than clade I, which has a mortality rate of 10–15% [28,29]. Clade I of the Mpox virus has been identified in several Central African nations, including the Democratic Republic of Congo, the Central African Republic, Kenya, Uganda, Rwanda, Malawi, and Tanzania, among others. In contrast, Clade II is prevalent across various countries on multiple continents, including Asia, Europe, Africa, and the Americas. Both clade I and clade II Mpox viruses have been detected in a range of countries, such as the USA, Canada, Brazil, France, Germany, Switzerland, the UK, Ireland, China, Pakistan, India, Thailand, South Africa, Oman, and Australia, among others [30] (Figure 2).

Since 2024, the majority of Mpox cases have continued to be reported from the African continent, largely driven by outbreaks of Mpox virus Ib in East African countries, including the DRC, where clade Ia is co-circulating. Sierra Leone, however, is experiencing a rapidly evolving outbreak that, based on available genomic sequencing results, appears to be driven by the Mpox virus clade IIb. Outside the African region, most cases reflect ongoing circulation of Mpox clade IIb among men who have sex with men (MSM) [6,31].

A significant factor influencing the evolution of the Mpox virus is the recombination between different strains of Mpox and other orthopoxviruses, such as the vaccinia virus. This recombination may enhance the Mpox virus’s ability to evade immune responses or adapt to new hosts. Additionally, compromises between human adaptation and the retention of zoonotic capabilities are evident in gene loss or pseudogenization, as seen with the D10L gene in Clade IIb [28,32].

Although Mpox is a DNA virus with a relatively stable genome, prolonged human transmission has led to a surge in its substitution rate in Clade IIb, up to 6–12 times that of strains before 2018. This acceleration underscores the importance of human reservoirs in viral development, marking a departure from historical trends [32,33].

### 3.2. Genomic Characteristics of Mpox Virus

The genome of the Mpox virus consists of approximately 197 kilobases of linear, double-stranded DNA, characterised by covalently closed hairpin ends and inverted terminal repeats (ITRs) of approximately 10 kilobases at both termini. It encodes a total of 181 proteins. The central conserved region contains housekeeping genes crucial for replication, transcription, and virion assembly, while genes at the ends are variable and play roles in the virus’s pathogenesis and host range [34]. The central region remains relatively conserved across Mpox virus strains and shows over 90% identity with analogous genes in other orthopoxviruses [31,32]. In contrast, the more variable terminal regions include genes linked to pathogenesis and host range, which facilitate modulation of the immune response and the development of species-specific characteristics, and show significant variation among different strains of Mpox virus [35]. Within the ITR region of the Mpox virus, there are at least four open reading frames (ORFs) [36], which may contribute to maintaining genome stability and influencing evolutionary processes [35].

The central genomic regions of the Mpox virus genome contain the open reading frames (ORFs) *A25R* and *C10L*, which exhibit a 96.3% similarity to the corresponding regions in the smallpox virus [37,38]. The Mpox virus genome encodes all the proteins necessary for DNA replication, including the helicase-primase E5, which is vital for genome uncoating and DNA replication [39]. The F8–A22–E4 polymerase holoenzyme is crucial for the accurate replication of the viral genome. Unlike other DNA polymerases, this holoenzyme incorporates a multifunctional cofactor, A22–E4, which is involved in processivity, proofreading, and repair during replication [40]. After replication occurs in the cytoplasm, the virally encoded RNA polymerase generates two distinct infectious forms i.e., the extracellular enveloped virus (EEV), which exits the cell through exocytosis and possesses a lipid envelope derived from the Golgi complex or endosomes, and the intracellular mature virus (IMV), which is released upon cell lysis and features a robust lipoprotein membrane that facilitates its spread between animals [41].

The Mpox virus genome encodes a variety of proteins critical to viral pathogenesis [42]. The conserved central genomic region encodes structural proteins that appear in various forms within virions. The enveloped extracellular virus (EEV) form generates membrane proteins such as C19L, A35R, and B6R, while the intracellular mature virus (IMV) form produces proteins including A29, M1R, E8L, H3L, and L1R. Among these, proteins such as A35 are vital for virulence, whereas L1R plays a key role in facilitating viral entry, highlighting their roles in cellular invasion, viral transmission, and pathogenicity [42]. The A29L protein, a homolog of the vaccinia virus A27L protein, is crucial for the virus’s attachment to host cell membranes. Consequently, A29L is considered a promising target for diagnostics and a potential vaccine candidate, capable of eliciting neutralising antibodies and robust immune responses [43]. The *A35R* gene encodes the A35R protein, which is crucial for the virus’s pathogenicity; its absence results in significant attenuation [42]. In a study examining eight different host-derived cell lines, it was determined that while the A35 protein is not necessary for viral replication, its removal from the viral genome resulted in a 1000-fold reduction in virulence in a mouse model [44].

### 3.3. Global Epidemiology of Mpox

The discovery of Mpox infection dates back to the summer and fall of 1958, marked by two consecutive outbreaks of a skin disease resembling smallpox among captive cynomolgus monkeys used for polio vaccine research at Statens Serum Institut in Copenhagen, Denmark [45]. This was followed by similar occurrences at research institutes in The Netherlands, the United States, and France [46,47]. According to reports from the WHO, several countries in the rainforests of West and Central Africa, particularly within the Congo Basin, are recognised as endemic areas for Mpox. Here, the zoonotic transmission of the Mpox virus occurs among small rodents and various primates [20,48,49]. Over time, facilitated by advancements in global transport and trade, this infection has spread beyond African borders, leading to sporadic outbreaks and epidemics that pose a significant threat to healthcare systems and humanity.

#### 3.3.1. Epidemiology of Mpox Within the African Continent

The first reported case of Mpox transmitted from animals to humans occurred in a nine-month-old child in the Democratic Republic of the Congo (DRC) in 1970. The child was admitted to a hospital, displaying centrifugal rashes and clinical symptoms resembling those of smallpox [50]. Shortly after this report and the subsequent virological confirmation by the WHO, cases of Mpox were diagnosed both virologically and serologically in various countries within the African Congo basin and West Africa, including Liberia, Nigeria, and Sierra Leone, between 1970 and 1971 [17,51]. The infected populations were predominantly children who had close contact with wild primates or monkeys, which are believed to be the primary intermediate hosts for Mpox virus transmission [52]. The cessation of the global smallpox vaccination campaign, following the complete eradication of smallpox, led to a sudden increase in Mpox incidences, with 47 reported cases in Central and West Africa from 1970 to 1979 [53,54]. Notably, 38 of these cases occurred in the DRC, and 12 additional cases were reported in the DRC and other Central and West African countries between 1980 and 1981. Of those infected, 80% were unvaccinated children under the age of 10, with eight deaths (17%) reported among children aged between 7 months and 7 years [17,55]. In response to the rise in Mpox cases, an active surveillance program was initiated in the DRC to monitor the spread of the virus, ensure accurate diagnosis, and implement preventive measures. This effort led to identifying a significant number of cases-specifically, 386 new Mpox infections in the DRC alone, compared to just 18 cases in other Central and West African countries, with a notably higher proportion of infected children than adults [17,54,56,57]. However, in 1986, Mpox surveillance programs in the DRC were discontinued due to the widespread HIV epidemic across Africa, which led the WHO to shift its resources away from Mpox [58]. As a result, routine Mpox diagnosis was discontinued, leading to a decline in reported cases, ultimately falling to zero by the 1980s. Reported Mpox cases remained very low, nearly zero, in the DRC and other countries in Central and West Africa until 1996, when a cluster of 344 new cases was identified among an unvaccinated population in the DRC between 1996 and 1997 [59]. This resurgence brought global attention to Mpox in Africa, with cases eventually rising to 500 by 1999. Between 2001 and 2004, 136 suspected cases were reported in the DRC, of which 51 were later confirmed as Mpox-positive. Following that period, thousands of new cases were reported annually in the DRC until 2005 [60]. Other countries in West and Central Africa reported infrequent Mpox cases, likely due to a decline in the disease diagnosis [52]. Between 2010 and 2018, several African countries experienced outbreaks of Mpox, including the Democratic Republic of Congo, Cameroon, Liberia, the Central African Republic, Sierra Leone, the Ivory Coast, and Nigeria. In 2017, Nigeria reported an outbreak with 122 confirmed cases of Mpox across 17 states within a year, resulting in six confirmed deaths [61,62,63]. The incidence of Mpox fluctuated in endemic regions across the continent, with sporadic outbreaks attributed to irregular Mpox diagnoses, weakened healthcare facilities, other infectious diseases, and inadequate surveillance systems.

#### 3.3.2. Epidemiology of Mpox Outside of the African Continent

The first known case of Mpox outside the endemic region of Africa was identified in May 2003 in a 3-year-old girl from Wisconsin, USA, who was hospitalised due to cellulitis in her hand resulting from a bite by her pet prairie dog [64]. Subsequently, additional laboratory-confirmed cases of Mpox were reported in several states, including Illinois, Missouri, Ohio, and Indiana. These cases were suspected to have arisen from scratches or bites from pet prairie dogs imported along with Mpox-infected Gambian rats from Ghana, West Africa [65,66]. In total, 47 laboratory-diagnosed or suspected cases were reported that year across various states in the USA. Fortunately, no deaths occurred, and there was no evidence of direct human-to-human transmission [67]. Between 2018 and 2021, both virologically confirmed and suspected cases of Mpox were reported in the United States, the United Kingdom, Israel, and Singapore [68,69,70,71]. This spread was largely attributed to travellers returning from Africa, especially Nigeria, where an ongoing outbreak was ongoing [72]. The infected individuals received prompt medical attention, which halted the spread of Mpox [60]. However, Mpox transmission did not end there, leading to an alarming global outbreak in 2022.

#### 3.3.3. Global Outbreak of Mpox in 2022–2025

In 2022, the world experienced a global outbreak of Mpox, first highlighted by the UK Health Security Agency and the WHO after a human Mpox infection was diagnosed in a traveller returning to the UK from Nigeria in May [73]. Shortly thereafter, Italy and Portugal also confirmed their first cases of Mpox in May [74]. By the end of May 2022, the UK had reported 86 laboratory-confirmed cases linked to the initial case [75]. Mpox spreads from person to person through direct contact, with new cases continuing to emerge across the UK, various European countries, and beyond. By the first week of October 2022, Mpox had been reported in approximately 100 non-endemic countries, according to the CDC, with outbreaks occurring in a range of nations across Europe, Asia, Australia, North America, and South America, as well as in various countries on the African continent where the disease is endemic [52,76]. Public health experts initially believed that direct person-to-person transmission through close proximity was the primary mode of transmission. Events such as popular festivals and saunas were considered significant sources of Mpox transmission, particularly in countries such as Germany and France [52,77]. However, subsequent research from Italy, Spain, and Germany revealed multiple Mpox cases where Mpox DNA was identified in human semen samples. This led to the conclusion that homosexual activities among men were a major route of transmission during the 2022 mass outbreak [78,79,80].

According to a 2023 report by the WHO, 96.6% of the reported cases were male, with 84.4% of these individuals participating in homosexual intercourse, predominantly aged between 18 and 44 years, indicating sexual transmission as a major route during the 2022 outbreak [81]. From 1 January 2022, to 30 September 2024, a total of 109,699 laboratory-confirmed Mpox cases and 236 deaths have been reported to the WHO from its 123 member states across all six regions. The top ten countries with the highest reported cases globally since the onset of the Mpox outbreaks in 2022 are as follows: the USA (34,063 cases), Brazil (12,722), Spain (8282), the Democratic Republic of the Congo (7095), France (4334), Colombia (4269), Mexico (4177), the UK (4100), Peru (3948), and Germany (3932). African countries, being the endemic region and a focal point for the Mpox outbreak, are facing a significantly worse situation than the rest of the world, with 11,724 confirmed cases and 57 deaths reported from 1 January 2022, to 20 October 2024, across 22 WHO member states in Africa [81].

As of 12 October 2025, a total of 59,213 laboratory-confirmed cases, including 245 deaths, have been reported to WHO. Regionally, from January 2022 to September 2025, the African Region reported 53,647 cases and 227 deaths, showing a notable 82% decrease in September 2025 compared to August. The Region of the Americas recorded 70,451 cases and 154 deaths, while the Western Pacific Region reported 6488 cases and 18 deaths, indicated 97% monthly decrease. Other WHO regions reported comparatively fewer cases, suggesting the outbreak is declining worldwide [81].

### 3.4. Transmission

**Figure 3:**
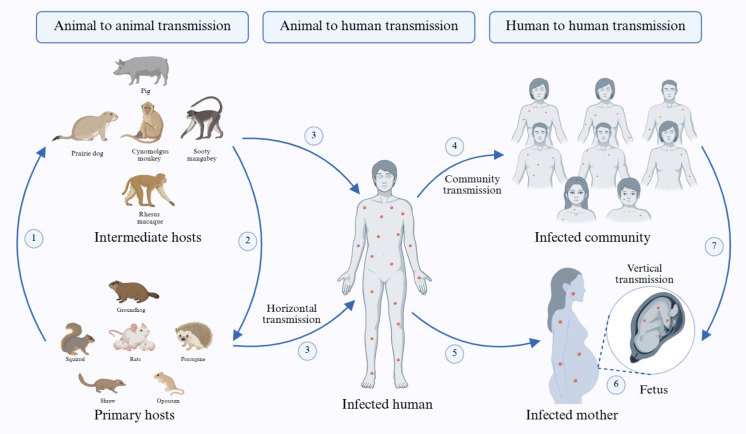
Schematic illustration showings the major sources of the Mpox virus and the potential routes of transmission. Steps: 1. Transmission of the Mpox virus from its infected primary hosts to the intermediate hosts; 2. Reverse transmission from the infected intermediate hosts to the primary hosts; 3. Horizontal transmission of the Mpox virus from infected animals to humans; 4. Transmission of the virus from infected individuals to the community; 5. Horizontal transmission of Mpox from an infected person to a pregnant woman; 6. Vertical transmission of the Mpox virus from the infected mother to the fetus; 7. Transmission of the Mpox virus to the pregnant woman from the infected individuals of the community. Created in BioRender. Adnan, N. (2026) https://BioRender.com/45zmjlr.

Although the name of the infection and its causative agent were derived initially from monkeys, the virus is believed to have its natural reservoir in various species of rodents and small primates inhabiting the forests of central Africa [82,83]. This zoonotic virus is transmitted from these animals, such as rats, squirrels, opossums, jerboas, elephant shrews, porcupines, and woodchucks, to different incidental intermediate hosts, typically primates including various species of macaques like rhesus and cynomolgus macaques, as well as monkeys like Sooty mangabey and Asian monkeys, domestic pigs, prairie dogs, and others, before ultimately reaching humans [84] (Figure 3). However, the exact natural reservoirs and the specific modes of animal-to-human transmission remain elusive [7,45]. Exposure to the virus can occur through various means, including direct contact with infected primary or intermediate hosts, bites or skin scratches, consumption of undercooked meat of infected animals, viral shedding from respiratory exudates or pustules, respiratory droplets, bodily fluids, particularly saliva or excrement of infected animals, and the international trade of exotic pets [57,85,86,87]. In the past, cases of Mpox were primarily associated with individuals who had been in contact with wild rodents and primates suspected of being infected with the virus, or with those who had returned from regions where Mpox is endemic [88,89]. However, in recent years, Mpox outbreaks have predominantly occurred through sexual contact with infected individuals [20]. Human-to-human transmission primarily occurs through direct contact with infected individuals, sharing personal belongings or food, and viral shedding from respiratory droplets, body fluids, and lesions [90,91,92,93]. Studies indicate that sexual activity is suspected as a mode of transmission in 95% of infected individuals, 98% of whom are either homosexual or bisexual [94,95]. Allan-Blitz et al. concluded that Mpox is a sexually transmitted disease due to the high consistency of its current transmission dynamics with those of STDs. Additionally, vertical transmission from mother to fetus may occur during delivery or close contact between mother and newborn during or after delivery [29,96,97,98] (Figure 3).

### 3.5. Pathogenesis

The respiratory route is the primary portal of entry for the Mpox virus, with the mucous membranes of the nasopharynx and oropharynx serving as its principal site of replication [37,99]. Additionally, Mpox may enter the body through various other routes, including the ocular, oral, rectal, genitourinary tracts, and subcutaneous portals [100]. The Mpox virus demonstrates broad host tissue and organ tropism, indicating its ability to effectively invade and utilise a wide range of animal reservoirs in non-endemic regions [101]. Various organs, such as the liver, kidneys, pancreas, ovaries, heart, lungs, eyes, and genitourinary organs, exhibit tissue tropism for the Mpox virus [102]. However, the specific mechanisms of cellular invasion and replication remain unknown. The tissue tropism of the Mpox virus is believed to be influenced by particular host–cell receptors, as well as factors such as the Mpox inhibitor of complement enzymes (MOPICE), complement control protein (CCP), and various viral ligands. These components play a role in the virus’s ability to bind host cell receptors, facilitating cell invasion and the initiation of replication. While the specific functions of these ligands remain elusive, it is suggested that the virus may employ a range of alternative ligands to interact with host cell receptors effectively [103]. Following successful entry of the mature virion, which consists of a single membrane, into the body, Mpox infects local cutaneous or mucosal cells, which serve as the primary replication sites for the virus [104,105]. This process releases various cytokines, ultimately attracting professional phagocytes, particularly macrophages and dendritic cells [17]. These phagocytic cells engulf the cells and the mature virion, allowing the virus to survive. This latent stage of Mpox pathogenesis is known as primary viremia, a period during which no symptoms or lesions are present and can last up to 2 weeks [74,106]. The Mpox virus is transmitted to local lymph nodes via phagocytic cells, leading to secondary viremia with symptoms such as atypical flu-like characteristics [37,107]. Following secondary viremia, the contagious prodromal stage begins as the virus spreads from the lymph nodes to other organs, such as the eyes, lungs, liver, and genitourinary organs, where it proliferates [18].

#### 3.5.1. Signs and Symptoms in Phases of Mpox Infection

Common signs seen in individuals infected with Mpox include rashes on various parts of the body, lesions in the anogenital and mucosal regions, fever, lethargy, muscle pain (myalgia), headaches, and lymphadenopathy (swelling of lymph nodes located in the neck, armpits, and groin). Additional symptoms may include sore throat, nasal congestion, cough, proctitis, tonsillitis, bacterial skin abscesses, ulcerated ventral tongue, genital vesicles, painful swallowing (odynophagia), perianal and tonsillar abscesses, rectal discomfort (which can lead to rectal perforation), and penile swelling [108]. Certain sexually transmitted infections (STIs), such as granuloma inguinale, molluscum contagiosum, chancres, and herpes simplex virus infections, can often be confused with Mpox [109].

The Mpox infection is generally divided into the invasive and cutaneous phases [110], and progresses through four distinct phases: incubation, prodromal, rash, and crusting [105] (Table 1).

##### Incubation Period

During the incubation period, the Mpox virus initially proliferates at the entry site, including the mouth, nose, throat, or damaged skin. It subsequently spreads to nearby lymph nodes, leading to viremia, and can also affect other tissues and organs. The incubation period typically lasts 6 to 13 days, but can range from 5 to 21 days in some cases, and often occurs without noticeable symptoms [105,111]. The onset of the illness is characterised by symptoms such as fever, chills, swollen lymph nodes (lymphadenopathy), headaches, back pain, muscle soreness, and fatigue (Table 1).

##### Prodromal Period

The prodromal phase lasts 1 to 4 days. It is characterised by fever, headaches, muscle pain, back discomfort, chills, fatigue, sore throat, difficulty breathing (dyspnea), and swollen lymph nodes, all occurring before rash onset. In rare cases, additional symptoms such as sweating and skin-related issues may occur [113]. Fever is the most common prodromal symptom, with body temperatures typically ranging from 101.3 °F (38.5 °C) to 104.9 °F (40.5 °C), peaking around the second day of fever [105] (Table 1).

##### Rash Period

The rash typically becomes visible 1 to 3 days after the onset of fever and spreads outward from the centre of the body. Initially, it appears as flat red spots that persist for 2 to 4 weeks before evolving into raised bumps, blisters, and, eventually, pus-filled lesions, crusts, and scabs [114]. The area’s most frequently affected by skin lesions include the face (97.5%), torso (92.5%), arms (87.5%), legs (85%), genitals (67.5%), scalp (62.5%), palms (55%), soles of the feet (50%), mouth (37.5%), and eyes (25%) [105]. These lesions crust over within 5 to 7 days and fall off within 7 to 14 days [115]. The resultant scars are often indented, resembling a naval, and may cause significant itching and discomfort. The number of lesions can vary widely, ranging from just a few to thousands. Lesion count classifies the severity of human Mpox: 1–25 as mild, 26–100 as moderate, 101–250 as severe, and over 250 as very severe [105] (Table 1).

##### Crusting Period

The pustules eventually develop crusts that change colour within one to two weeks [116]. The time from lesion appearance to scab shedding typically ranges from 2 to 4 weeks. The scabs that fall away may be significantly smaller than the original lesions, and erythema or pigmentation can persist even after the scabs have detached, often leaving more than half of the scar still visible [105]. In males, genital lesions may lead to complications such as paraphimosis and necrotic crusts. Lesions in the rectal area can cause proctitis, with pain during bowel movements being the primary symptom. Additionally, ulcers with central depressions may emerge on the oral mucosa, tongue, pharyngeal wall, and tonsils [112] (Table 1).

#### 3.5.2. Clinical Complications of Mpox Infection in Different Body Parts

##### Neurologic Complications

**Figure 4:**
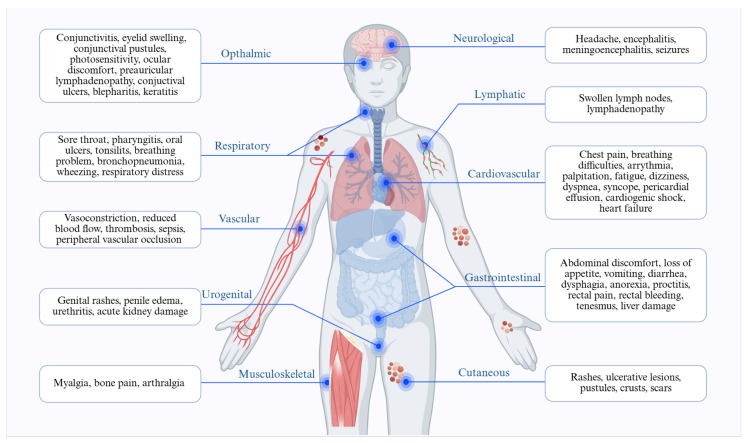
Schematic illustration outlining the major clinical manifestations and complications of Mpox. Created in BioRender. Adnan, N. (2026) https://BioRender.com/uek6dcu.

The most frequent neurological symptom is headache, often seen during the initial stage of the infection. Severe neurological issues, such as encephalitis, meningoencephalitis, and seizures, can arise after this initial phase [52] (Figure 4).

##### Ophthalmic Complications

The primary ophthalmic symptoms associated with Mpox infection include distinctive rashes and lesions, frontal headaches localised to the periorbital and orbital regions, conjunctivitis, and eyelid swelling [117,118]. The pustular rash, discharge, and crusted secretions may lead to an inability to open the eyelids for several days. Additionally, conjunctival pustules can develop, accompanied by excessive tearing, light sensitivity, and discomfort [118] (Figure 4). Kaufman et al. introduced the term Mpox-related ophthalmic disease (MPOXROD) to describe the range of eye-related manifestations that may occur with Mpox infection [119]. Ulcerative lesions and swollen eyelids typically appear at the margins of the eyelids and conjunctiva. Many patients have exhibited preauricular lymphadenopathy, along with vesicular and infiltrative lesions. Large conjunctival ulcers may have smooth edges and a pale colouration [119]. Symptoms related to pox-induced conjunctivitis can include conjunctival ulcers, pseudomembranous or subconjunctival nodules, extensive blistering, papular lesions on the conjunctiva, and follicular reactions of the conjunctiva. Secondary symptoms accompanying Mpox patients experiencing conjunctivitis include nausea, light sensitivity, mouth sores, swollen lymph nodes, sore throat, fatigue, chills, and sweating [117]. Blepharitis, characterised by inflammation of both eyelids, may develop due to a blockage in the small oil glands at the bases of the eyelashes, leading to irritation and redness [120].

##### Cardiovascular Complications

Cardiovascular effects associated with Mpox infection can vary in both type and severity. These effects may affect various aspects of the cardiovascular system, including arrhythmias, myocarditis, and pericarditis. Myocarditis can range from mild inflammation to severe heart muscle damage. The primary mechanism underlying Mpox-related myocarditis is lymphocytic inflammation, typically occurring 10 to 14 days after the initial infection, followed by myonecrosis [121,122]. Common symptoms include chest pain, breathing difficulties, irregular heartbeats, fatigue, and diminished exercise capacity. In severe cases, myocarditis can lead to acute heart failure, cardiogenic shock, or even sudden cardiac death [123].

Mpox can lead to pericarditis, an inflammation of the pericardium that often causes sharp chest pain, particularly when lying down or deep breathing. Additional symptoms may include a low-grade fever, cough, and a distinctive friction rub detectable during a chest examination. Symptoms associated with pericarditis encompass chest pain, fatigue, shortness of breath (dyspnea), palpitations, and, in severe cases, cardiogenic shock [124]. The inflamed pericardium often intensifies chest pain with deep breaths or when positioned horizontally. Moreover, in more critical scenarios, fluid accumulation in the pericardial space, known as pericardial effusion, may impair cardiac function [125] (Figure 4).

Mpox may also disrupt the heart’s regular electrical conduction system, leading to arrhythmias. During Mpox infection, symptoms of arrhythmia may include palpitations, dizziness or lightheadedness, fainting (syncope), and chest pain [123]. This interference can occur directly, disrupting the rhythm and timing of heartbeats [123]. Inflammation and damage to the heart muscle or its conduction pathways can disrupt electrical signals, resulting in irregular heart rhythms [125].

##### Gastrointestinal Complications

The gastrointestinal complications associated with Mpox infection may encompass abdominal discomfort, anorexia, diarrhoea, nausea, vomiting, proctitis, rectal pain, painful bowel movements, and the sensation of incomplete defecation (tenesmus) [126,127] (Figure 4). There have also been reported cases of liver damage linked to this condition. Loss of appetite is the most common gastrointestinal symptom of Mpox, affecting 47% of patients, as reported by Simadibrata et al. Other commonly reported symptoms include vomiting (12%), nausea (10%), abdominal discomfort (9%), and diarrhoea (5%). Additionally, proctitis, along with rectal or anal discomfort and rectal bleeding, is prevalent, with relative frequencies of 11%, 25%, and 12%, respectively [127].

##### Pulmonary Complications

The respiratory system may be affected, leading to severe coughing, difficulty breathing, or bronchopneumonia [108]. The impacted areas within the respiratory organs can be divided into three categories: upper respiratory (which includes symptoms such as a runny nose, sore throat, pharyngitis, oral ulcers, and tonsillitis), lower respiratory (characterized by wheezing, coughing, and respiratory distress), and general respiratory (which encompasses a combination of symptoms from both the upper and lower respiratory categories) [128] (Figure 4).

### 3.6. Diagnosis of Mpox

Mpox is primarily diagnosed based on a combination of clinical indicators and epidemiological factors, with definitive confirmation obtained by nucleic acid amplification testing (NAAT) [74]. The primary method for identifying suspected cases involves detecting viral DNA from swabs collected from the patient’s skin lesions and wounds. A comprehensive diagnosis should incorporate both thorough clinical evaluations and laboratory analyses [129,130,131]. A range of advanced laboratory techniques is currently utilised for Mpox diagnosis. Key methods include cell culture, polymerase chain reaction (PCR), and enzyme-linked immunosorbent assay (ELISA). Additionally, tests include immunohistochemistry, electron microscopy, virus isolation, culture and cytopathic effect (CPE) screening, immunofluorescent antibody testing, and in situ histopathological examination. Biosensors are cutting-edge tools designed for rapid viral detection, while Western blot analysis and genetic sequencing further characterise the virus’s structural and genetic properties [129,132,133,134,135,136].

To perform these diagnostic procedures, a variety of clinical samples are necessary, including fluid from lesions, dried scab material, crusts, oropharyngeal and nasopharyngeal swabs, tonsillar tissue, punch biopsy specimens, whole blood, and serum collected from both the acute and convalescent phases of infection. The most effective specimens for laboratory confirmation are those obtained directly from skin lesions. All samples that could potentially contain the Mpox virus should be processed in Biosafety Level 2 (BSL-2) facilities, which minimise exposure risks and safeguard the integrity of diagnostic testing [133].

#### 3.6.1. Polymerase Chain Reaction (PCR)

Real-time polymerase chain reaction (RT-PCR) is an advanced genetic technique used to detect the Mpox virus. This method emphasises amplifying specific target genes typically located within the conserved regions of the virus’s genetic material. Key genes selected for PCR amplification include the extracellular envelope protein gene (*B6R*), the *E9L* gene, which encodes DNA polymerase, and the *RPO18* gene, an essential subunit of DNA-dependent RNA polymerase. Additional genes of interest include the complement-binding protein genes, namely *C3L*, *F3L*, and *N3R* [109]. Both RT-PCR and conventional PCR tests must be conducted in a laboratory environment classified as Biosafety Level 2 (BSL-2) to ensure safety, given the virus’s infectious nature [137]. To further confirm the presence of the Mpox virus, PCR-amplified genes or gene fragments may be analysed using restriction length fragment polymorphism (RFLP) [134]. A positive result from an *orthopoxvirus* PCR test, followed by a confirmatory Mpox-specific PCR and sequencing, provides strong evidence of an active Mpox infection. Notably, the PCR test is recognised as the gold standard in laboratory examinations due to its outstanding specificity and sensitivity, making it essential for rapidly and accurately detecting the monkeypox virus [133,135,138,139] (Table 2).

#### 3.6.2. Immunologic Assays

Serological methods detect Mpox antigens or IgG and IgM antibodies using techniques such as immunohistochemistry, ELISA, and rapid tests such as lateral flow and dot blot assays. ELISA is the preferred method for serum antibody detection, with specific IgM appearing around 7 days and IgG around 21 days post-rash onset [143,146]. Immunochemistry analysis can employ either polyclonal or monoclonal antibodies targeting orthopoxviruses to differentiate between poxvirus and herpesvirus infections. Levels of antiviral antibodies and T-cell responses have been observed to increase around the time the illness manifests. If an individual without a history of rash or severe illness tests positive for both IgM and IgG antibodies, an indirect diagnosis of Mpox may be considered [134]. Serological testing for Mpox can reinforce a diagnosis, especially when NAAT is not feasible. Detecting IgM in acutely ill patients (within 4 to 56 days post-rash onset) or measuring IgG in paired serum samples (collected at least 21 days apart, with the first taken during the first week of illness) can further assist the diagnosis [74]. The ELISA can detect specific IgM and IgG antibodies in the serum of patients infected with Mpox approximately 5 and 8 days after infection, respectively, making it a valuable tool for serological testing. A fourfold increase in serum antibodies can be utilised to diagnose Mpox infection during both the acute and convalescent phases [109]. A CDC study analysed sera from 37 confirmed Mpox cases during the 2003 outbreak in the USA using IgM-capture and IgG ELISAs targeting *orthopoxvirus* antigens. It detected IgM in 94.5% of cases (optimal ≥5 days after rash onset) and IgG in 80.5% (optimal ≥8 days), with high sensitivity, e.g., 94.8% for IgM and 100% for IgG, and good specificity of 94.5% for IgM and 88.5% for IgG, correlating well with PCR [155]. Various immunological methods are available for detecting IgG and IgM antibodies, as well as immunohistochemistry for identifying viral antigens to detect orthopoxvirus DNA. However, due to the serological cross-reactivity of orthopoxviruses, none of these tests can provide specific confirmation of Mpox, as this phenomenon was seen in the case of the detection assay of other viruses [156] (Table 2).

#### 3.6.3. Electron Microscopy

Electron microscopy is a crucial tool for detecting poxvirus infections and is one of the early indicators of rash-related illnesses. When examined under an electron microscope, the distinctive morphology of poxvirus virions becomes apparent. A notable instance of this was observed during the recent Mpox outbreak in the United States, where electron imaging revealed keratinocytes containing a significant number of mature virions alongside immature virions in the synthesis phase, often referred to as “viral factories”, situated within the cytoplasm of infected cells [134,140,141]. However, relying exclusively on electron microscopy for poxvirus identification is not entirely reliable, as virions cannot be definitively identified by their shape alone [109], and it is important to distinguish the Mpox virus from other virus families, such as Herpes and Parapox viruses [133,142]. Moreover, the need for a complex laboratory setup, specialised, trained personnel, specific reagents, and high operating and maintenance costs limits its practicality and usefulness as a general diagnostic method for Mpox. Therefore, it can serve as an additional technique to support other diagnostic methods [142,157] (Table 2).

#### 3.6.4. Other Diagnostic Approaches

Multiple nucleic acid amplification tests have been developed to identify Mpox. Combining two distinct viral gene targets, such as *E9L* and *B6R*, can provide a reliable, sensitive, and rapid diagnostic method. However, access to these tests remains limited. Recently, a self-contained cartridge, the Cepheid GeneXpert, was developed as an alternative to traditional PCR detection methods. The GeneXpert assay has demonstrated high sensitivity, specificity, negative predictive value, and positive predictive value in suspected specimens, regardless of specimen type (crust or vesicular swab) [154,158]. As with electron microscopy, the cultivation of viruses for detection poses various limitations and challenges. Key among these are the need to identify suitable cells for virus replication, isolate the virus, and have a laboratory equipped with the necessary tools. However, cytopathic effect (CPE) screening methods are employed for certain viruses. Research has shown that the Mpox virus replicates rapidly in cell cultures, displaying detectable CPE in Rhesus Monkey Kidney (RMK), Buffalo Green Monkey (BGM), A549, and Medical Research Council cell strain 5 (MRC-5) cell lines within approximately two days. Despite this, cell culture and electron microscopy techniques remain critical in vaccine preparation, but not for diagnostics. Today, viral cultures have primarily been replaced by nucleic acid amplification tests and antigen detection methods [129]. Individuals infected with Mpox typically exhibit detectable levels of anti-*orthopoxvirus* antibodies. Skin tissue biopsies can also serve as viable clinical samples for diagnostic evaluation when clinically indicated [74]. Furthermore, various methods, including recombinase polymerase amplification (RPA), CRISPR-Cas-based technique, loop-mediated isothermal amplification (LAMP), and restriction length fragment polymorphism (RFLP), have been developed to detect Mpox virus DNA [109,144,145,147,148,149,150,151] (Table 2). The World Health Organisation recommends nucleic acid amplification testing (NAAT) for detecting the Mpox viral genome, hemagglutinin, acidophilic-type inclusion bodies, and the *crmB* genes [152,159]. Immunohistochemical examination with monoclonal and polyclonal antibodies can aid in distinguishing a herpesvirus infection from a poxvirus disease [134,146].

A flexible lateral flow immunoassay (LFIA) has been developed, featuring robust colourimetric capabilities and enhanced dual-signal fluorescence output. This innovation enables rapid, on-site, highly sensitive detection of the Mpox virus antigen (A29L) [160]. Utilising readily accessible materials and demonstrating no cross-reactivity, an electrochemical biosensor based on electrochemical impedance spectroscopy (EIS) has been shown to effectively identify the A29 viral protein from Mpox virus antigens in saliva and plasma samples within 15 min [161]. The MAGLUMI^®^ Mpox virus Ag Chemiluminescence Immunoassay (CLIA) assay kit, produced by Shenzhen New Industries Biomedical Engineering Co., Ltd. (Snibe), employs a double-Mpox virus monoclonal antibody that selectively targets the A29 and A35R epitopes [162]. This represents the first application of the Helicase-Dependent Amplification (HDA) method, combined with a lateral flow test (LFT), to detect Mpox infection. The HDA-LFT assay exhibited 94% specificity and 86% sensitivity in identifying the Mpox virus-specific conserved fragment F3L [163]. Additionally, a highly sensitive and specific MPXV-MCDA-LFB assay was developed, integrating isothermal multiple cross-displacement amplification (MCDA) with a nanoparticle-based lateral flow biosensor (LFB) for the rapid detection of Mpox virus target genes (*D14L* and *ATI*). This assay demonstrated no cross-reactivity with non-Mpox virus templates, establishing it as a reliable diagnostic tool for Mpox infection [164].

### 3.7. Treatment Approaches and Medications for Mpox

#### 3.7.1. Supportive Care and Conventional Therapeutic Supports

The management of Mpox has integrated supportive care with targeted antiviral therapies and vaccination strategies, especially following the 2022 outbreaks [165]. In most cases, supportive care is sufficient to meet patients’ clinical needs. Since there are no Food and Drug Administration (FDA)-approved antivirals outside of special circumstances, the main focus of treatment is symptom management. Although many antiviral agents are in clinical trials or used under compassionate use protocols, none are FDA-approved for Mpox [29,166]. On the other hand, for mild infections, symptomatic treatment is recommended to relieve symptoms; however, for severe Mpox, the focus shifts to supportive care to manage systemic complications, including secondary bacterial infections [167].

Therapeutic interventions may include administering antipyretics to reduce fever and flu-like symptoms, analgesics for pain relief, and antibiotics when secondary infections occur. Additionally, treatment should be customised to meet the specific needs of vulnerable groups, such as individuals with serious health conditions, immunocompromised patients, people living with HIV, as well as pregnant women, children, and the elderly [29].

#### 3.7.2. Antiviral Drugs to Treat Mpox

It is essential to note that antiviral medications specifically approved for the treatment of Mpox have not yet received final authorisation [168,169]. Several studies are currently underway to evaluate the effectiveness of treatment approaches and therapies traditionally used for smallpox in managing Mpox [37,170]. The role of antibody responses in protecting individuals against infections caused by poxviruses has been documented [171]. Several antiviral agents, including tecovirimat, cidofovir, and brincidofovir, originally developed for the treatment of smallpox and related infections caused by other orthopoxviruses, are now being repurposed for Mpox [129,172,173,174,175,176]. The Centres for Disease Control and Prevention (CDC) has implemented a new protocol permitting the emergency use of tecovirimat for non-variola infections, including those diagnosed with Mpox. However, there is limited evidence supporting the effectiveness of cidofovir and, even less so, tecovirimat in treating severe cases of Mpox [177,178,179].

Cidofovir has been utilised in various studies as a treatment option for severe cases of Mpox, albeit with a limited patient cohort. Research conducted by Raccagni et al. reported no documented side effects or significant changes in creatinine levels following cidofovir administration. Additionally, the article indicated that patients did not exhibit any new symptoms after treatment [180]. Tecovirimat has been used in a randomised controlled trial (RCT) for endemic Mpox (Clade I MPXV) and in a phase-3, double-blinded RCT for Clade II mpox. However, the results did not show significance for early clinical resolution of lesions, pain reduction, or viral clearance among participants [181,182]. Immunotherapy for Mpox shows promise in mitigating the severity of the disease. The potential benefits of combining antiviral medications with immunotherapy may exceed those achieved through antiviral treatment alone, thereby enhancing overall therapeutic efficacy [183,184,185].

##### Mechanism of Actions of Antiviral Drugs Against Mpox

**Figure 5:**
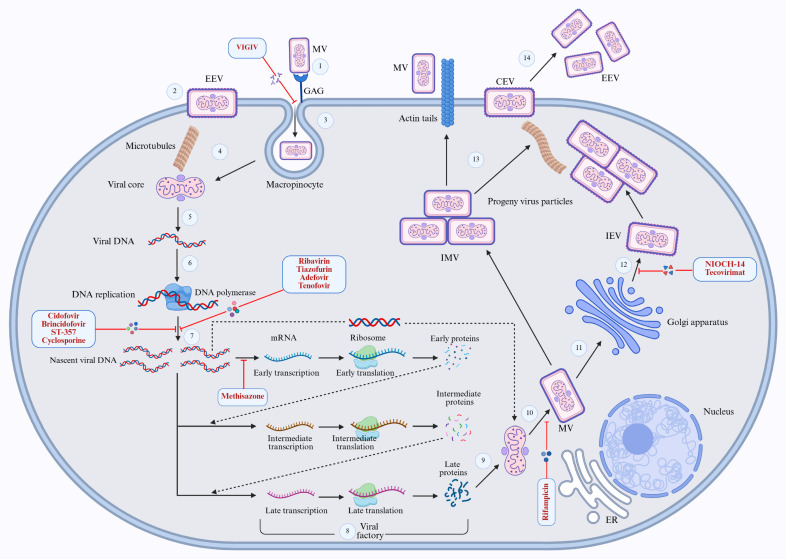
Pathogenesis of Mpox and action mechanisms of the major antiviral drugs used to treat Mpox. Steps: 1. Attachment of MV with the host cell surface GAG receptor; 2. Fusion of EEV with the host cell membrane; 3. GAG receptor mediated macropinocytosis of the receptor-bound MV; 4. Microtubular transport and subsequent uncoating of the virus particles to release the viral core into the host cell cytoplasm; 5. Release of the double-stranded DNA into the cytoplasm; 6. Attachment of DNA polymerase enzyme to the viral dsDNA; 7. Replication of the dsDNA to produce nascent dsDNA; 8. Synthesis of the early, intermediate, and late proteins from the dsDNA in the viral factory; 9. Assembly of the proteins and the nascent dsDNAs for viral core formation; 10. Formation of MVs through the assembly of the virus components; 11. Maturation of the newly synthesized virus particles using trans-Golgi apparatus to form IEV; 12. Formation of the progeny IMVs and IEVs in the cell cytoplasm; 13. Transport of the progeny IMVs and IEVs to the periphery of the host cell through actin efflux or microtubular system; 14. Release of the progeny virus particles outside of the host cell. Antiviral drugs for Mpox target critical steps in the viral life cycle, including viral entry, DNA replication, and protein synthesis. Drugs such as cidofovir, brincidofovir, ST-357, cyclosporine, ribavirin, tiazofurin, adefovir, and tenofovir inhibit DNA polymerase. Others, including tecovirimat and NIOCH-14, hinder virus envelopment, preventing virion release. Intravenous immunoglobulin, such as VIGIV, neutralizes viruses and blocks entry. Created in BioRender. Adnan, N. (2026) https://BioRender.com/yf22w1v. Abbreviations: MV—mature virion; EEV—extracellular enveloped virion; CEV—cell associated enveloped virion; IEV—intracellular enveloped virion; IMV—intracellular mature virion; GAG—glycosaminoglycan; VIGV—vaccinia immune globulin intravenous; mRNA—messenger ribonucleic acid; ER—endoplasmic reticulum.

Multiple authorised antiviral medications such as cidofovir, brincidofovir, and tecovirimat have been developed to target various orthopoxviruses, particularly smallpox [186]. These drugs are used to treat poxvirus infections, especially during outbreaks, due to their demonstrated potent antiviral activity against various orthopoxviruses, including smallpox, camelpox, cowpox, molluscipox, parapox, and Mpox [34]. Cidofovir, also known as CDV, is an acyclic nucleoside phosphonate (ANP) initially approved by the FDA for the treatment of cytomegalovirus (CMV) retinitis in patients with HIV [187,188]. This prodrug requires phosphorylation to become active in the cell cytoplasm via cellular enzymatic activities and has demonstrated efficacy against various poxviruses in multiple animal model clinical trials [188,189]. Functioning as a modified cytosine, the drug blocks the viral DNA polymerase by preventing template DNA extension during viral replication. It also disrupts the 3′-5′ exonuclease activity of the DNA polymerase during the DNA proofreading step. It ultimately inhibits the synthesis of the viral E9 enzyme, which is essential for the viral replication process [190,191] (Figure 5).

Another orally administrable antiviral prodrug with the exact mechanism of action was developed by covalently conjugating a lipophilic alcohol moiety to cidofovir to enhance its cellular activity and conversion to its active form via cellular enzymes [192]. This antiviral drug, known as brincidofovir, underwent multiple clinical trials involving different phages and demonstrated significant antiviral activity against double-stranded DNA viruses, particularly the smallpox virus. It received FDA approval for the treatment of smallpox [193,194]. Tecovirimat, a viral protein synthesis inhibitor, stands out as a notable antiviral drug authorised by the FDA for the treatment of smallpox. Derived from 4-trifluoromethyl phenol, this drug demonstrates antiviral activity against poxviruses by targeting the synthesis of viral protein VP37, which is necessary to envelop mature virus particles within the Golgi apparatus [195]. Consequently, tecovirimat inhibits the release of viruses from infected cells, as only enveloped viral particles can exit host cells [196,197] (Figure 5) (Table 3).

The Russian VECTOR developed another VP37 synthesis inhibitor, Nioch-14, which showed significant antiviral activity against poxviruses, comparable to tecovirimat. This drug is a small-molecule inhibitor derived from pyrroledione and is orally bioavailable [198,199]. A newly synthesised drug, designated ST-357, showed promising antiviral activity against vaccinia and cowpox viruses. It is intended for use as a second-line treatment against Mpox and smallpox. The mechanism of action of this compound is entirely different from those of cidofovir, brincidofovir, and tecovirimat. It works by inhibiting the poly-A polymerase enzyme activity of the viruses, which is essential for DNA replication [200] (Figure 5) (Table 3).

##### Newer Antiviral Drug Candidates

A diverse range of synthetic and semi-synthetic compounds with demonstrated antiviral efficacy against various orthopoxviruses are undergoing development or clinical trials. Several nucleoside-based antiviral compounds, such as ribavirin, tiazofurin, adefovir, and tenofovir, have shown significant potential to reduce the replication of poxviruses by inhibiting the synthesis of inosine monophosphate dehydrogenase (IMP) [193,201] (Figure 5). C-CA3-ADO and C3-NPC A compounds are capable of inhibiting the synthesis of S-adenosylhomocysteine hydrolase, while HPMA and Adenosine N1 oxide (ANO) function as DNA polymerase inhibitors to impede DNA replication [178]. Various antibiotics and chemotherapeutic compounds, including novobiocin, coumermycin, nigericin, distamycin, and mitoxantrone, have shown antivaccinia activity. Additionally, thiosemicarbazones such as marboran and methisazone can inhibit the transcription of viral mRNA in orthopoxviruses. Tetrapyrroles, aptamers, and phospholipid compounds have also demonstrated inhibitory activities against vaccinia and various orthopoxviruses, indicating their potential as secondary defence drugs against Mpox and smallpox [193,201] (Figure 5).

#### 3.7.3. Antimicrobial Drugs to Treat Mpox

Conventional antibiotics, cyclosporine, and rifampicin are being used to some extent to treat Mpox. Cyclosporine inhibits poxvirus DNA replication by incorporating into the nascent DNA chain [194]. In contrast, the rifampicin antibiotic, known to be effective against poxviruses for a long time, exerts its antiviral activity against poxviruses by blocking the interaction between the viral proteins D13 and A17, which is necessary for the synthesis, assembly, and maturation of membrane precursor proteins [193,202] (Figure 5).

In addition to these drugs, immunoglobulin therapy, such as Vaccinia Immune Globulin Intravenous (VIGIV), is approved by the FDA and recommended by the CDC [203]. It is administered as an intravenous immunoglobulin solution containing high titers of IgG against vaccinia virus derived from recovered patients. This therapy is used to manage complications, such as severe generalised vaccinia, following smallpox vaccination [60,204] (Figure 5).

### 3.8. Vaccines Against Mpox

Currently, no vaccine specifically designed for Mpox is available, necessitating reliance on vaccinia virus-based vaccines, initially developed for smallpox, since members of the *Orthopoxvirus* genus are capable of inducing serological cross-protection against Mpox [205,206]. The history of smallpox vaccination dates back to the use of bovine pox pustules, long before the discovery of viruses and the development of modern immunology and vaccination methods [207]. Over time, this approach evolved to include the use of attenuated viral strains, leading to the development of safer, more effective vaccines, driven by advancements in virology, immunology, and vaccine research.

#### 3.8.1. Traditional Mpox Vaccine Candidates

Traditional Mpox vaccine candidates, ranging from inactivated whole-cell vaccines to recent attenuated vaccines, have been developed, approved, and administered in different countries to provide immunity against various orthopoxviruses [208,209]. Attenuated strain-based vaccines can be categorised into three generations based on the attenuation technique, cultivation methods, and replication status of the vaccine strains [210,211].

The initial smallpox vaccine, developed in the previous century, utilised live vaccinia virus strains such as Lister/Elstree, EM-63, or Tian-Tian. These strains were obtained from calf lymph nodes, purified, and lyophilised before being administered by scarification using bifurcated needles [210]. The Dryvax vaccine, developed and administered in the USA, is a first-generation, live, replication-competent vaccinia virus-based vaccine that offers protection against both smallpox and Mpox [212,213,214,215] (Table 4). While demonstrating notable effectiveness against Mpox, the vaccine was associated with occasional severe adverse effects, including Stevens-Johnson syndrome, myo/pericarditis, fetal vaccinia, encephalitis, progressive vaccinia, eczema vaccinatum, and occasional fatalities among patients, particularly among the immunocompromised [216].

**Figure 6:**
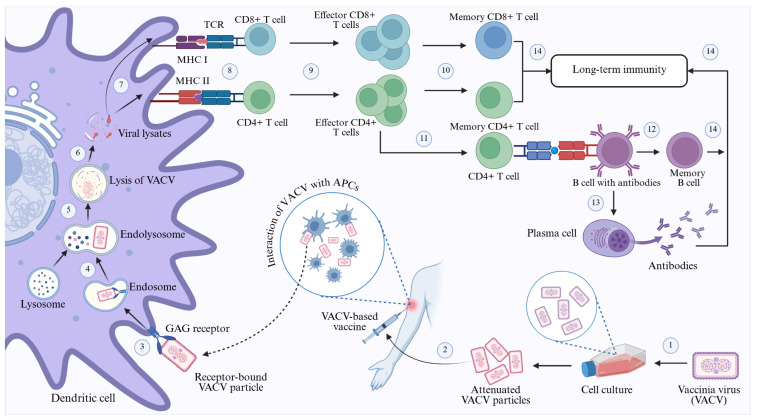
Mechanism of action of the attenuated Vaccinia virus-based vaccine used for immunisation against Mpox. Steps: 1. Attenuation of live VACV particles through repeated cell culture; 2. Preparation and administration of VACV-based vaccine; 3. Attachment and subsequent entry of VACV particles into dendritic cells through GAG receptor-mediated macropinocytosis; 4. Formation of the endolysosome through fusion of the VACV-containing endosome with lysosome; 5. Endosomal lysis of VACV particles by lysozyme; 6. Release of the VACV lysates into the cytoplasm of the dendritic cell; 7. Presentation of the antigenic viral lysates on the dendritic cell through the interaction with MHC class I and MHC class II molecules; 8. Attachment of CD8+ and CD4+ T cells with antigen-bound MHC I and MHC II receptors, respectively, through their interaction with T cell receptors; 9. Activation of the CD4+ and CD8+ T cells and production of the effector T cells; 10. Generation of memory CD4+ and CD8+ T cells from the effector cells; 11. Attachment and CD4+ mediated activation of B cells; 12. Conversion of effector B cell into memory B cell; 13. Generation of antibody-secreting plasma cells from the effector B cells; 14. Memory T cells, memory B cells, and plasma cells provide long-term immunity. Created in BioRender. Adnan, N. (2026) https://BioRender.com/exe6ksk. Abbreviations: VACV—vaccinia virus; APC—antigen-presenting cell; GAG—glycosaminoglycan; MHC—major histocompatibility complex; TCR—T cell receptor; CD—cluster of differentiation.

The second-generation vaccines include several different live attenuated vaccines, such as the Lister-based vaccine (RIVM), Elstree-BN, the Aventis Pasteur Smallpox Vaccine (APSV), the vaccinia virus-based Lister vaccine, and the advanced live attenuated vaccine ACAM2000 [218,219,230]. The Lister-based vaccine (RIVM) is a single-passage, live-attenuated, replication-competent vaccinia virus derived from strains isolated from calf lymph nodes and directly transferred to rabbit kidney cells for replication [231]. The Elstree-BN smallpox vaccine, a second-generation vaccine, was developed using the Lister strain, replicated in chicken embryonic cells [232]. Additionally, the ACAM2000 vaccine, derived from Dryvax, demonstrated greater immunogenicity and fewer adverse effects than first-generation vaccines [220,221,222,233,234] (Figure 6) (Table 4).

Additionally, the Aventis Pasteur smallpox vaccine, a second-generation vaccine based on replication-competent vaccinia virus, exhibits safety and immunogenic properties similar to those of ACAM2000 [217,235]. Although effective against smallpox, this vaccine has not yet been used for Mpox.

The third-generation live attenuated smallpox vaccines were developed using replication-deficient, modified vaccinia virus strains to mitigate adverse effects stemming from contamination with animal lymph nodes or complications arising from autoinoculation during vaccine strain development. The LC16m8 vaccine, a representative of third-generation attenuated vaccines, was developed in Japan in 1970. The vaccine was derived from the Lister strain, which underwent passage in primary rabbit kidney cells and Vero cells. chorioallantoic membrane (CAM) to obtain the desired clone, designated LC16m8. This strain exhibited complete non-replicating and non-infectious characteristics, rendering it suitable for use in the vaccine [223,224,225,226,236]. The recently developed live attenuated vaccine, IMVAMUNE, also known as JYNNEOS or IMVANEX, represents a cutting-edge, cell culture-based, third-generation smallpox vaccine. This advanced vaccine was developed using a modified vaccinia Ankara strain that underwent 596 passages in chick embryo fibroblasts and was subsequently attenuated through multiple rounds of dilution. IMVAMUNE, is authorised by the Advisory Committee on Immunisation Practices (ACIP) and the US FDA for pre-exposure prophylaxis against smallpox and Mpox [237,238,239] (Table 4).

Upon administration, the attenuated strains, combined with vaccine adjuvants, promote the uptake of specific viral surface antigens by dendritic cells at the vaccination site. Subsequently, these dendritic cells are activated by pattern recognition receptors in response to cytokine signals generated by the adjuvants. Upon recognition by dendritic cells, the vaccine strains’ antigens are transported to the lymph nodes, where they are presented to T cells by MHC I and MHC II molecules. Subsequently, T cells are activated by binding to the antigen-presenting MHC molecules through their T cell receptors. The MHC class I molecule activates CD8+ T cells, assisted by helper T cells, which initiate cytotoxic activity and generate memory T cells. Conversely, when presenting antigens, the MHC class II molecule stimulates CD4+ T cells, also known as helper T cells, by releasing cytokines. This activation of CD4+ T cells subsequently triggers B cells to differentiate into plasma cells, thereby increasing antibody production and forming memory B cells. The resulting memory B and T cells persist in the body, conferring long-lasting immunity against the antigens and orchestrating a response to neutralise or eliminate poxviruses upon re-entry [240,241] (Figure 6).

#### 3.8.2. Novel Mpox Vaccine Candidates

Multiple novel Mpox vaccine candidates, including protein subunit, recombinant protein subunit, virus-like particle (VLP)- based, and nucleic acid-based (e.g., DNA and mRNA) vaccines, have been designed and are currently undergoing in silico, molecular docking, or animal trials [230]. The novel vaccine platforms were designed to boost both humoral and cellular immune responses while reducing the likelihood of adverse side effects, such as reactogenicity. Additionally, they aim to minimise labour, expenses, and, most importantly, the risks associated with developing traditional live-virus-based vaccines [242]. Several protein-subunit vaccines have been developed utilising distinct immunogenic glycoprotein fragments of the vaccinia virus, combined with various adjuvants to augment immune responses [243]. Notably, Buchman et al. detailed the design of a protein-based smallpox vaccine. This vaccine incorporates purified ectodomains of A33 and B5, extracted from the enveloped vaccinia virion, and L1 and A27 proteins from the mature vaccinia virion. Furthermore, the vaccine formulation included aluminium hydroxide and CpG as adjuvants. The immunogenicity and safety profile of this vaccine were assessed through animal model studies involving cynomolgus macaques [244].

Bhattacharya et al. have outlined a novel multi-epitopic peptide-based potential vaccine candidate against Mpox. This vaccine incorporates 10 B- and T-cell-derived epitopes spanning 147 amino acid residues. Additionally, the CTxB adjuvant is attached to the N-terminus via the EAAAK peptide linker, and the PADRE sequence (AKFVAAWTLKAAA) is attached to the C-terminus to enhance the vaccine’s immunogenicity [245].

Shantier et al. developed a 275-amino-acid, multi-epitopic protein subunit vaccine. This vaccine was constructed using MHC-1 and MHC-2 molecules, B cell epitopes, appropriate linkers (e.g., EAAAK), and adjuvants, including the PADRE sequence and RS09 [246]. Similarly, Heraud et al. designed a subunit recombinant vaccine using plasmid DNA encoding the Mpox orthologs of the vaccine virus proteins L1R, A27L, A33R, and B5R. This vaccine has demonstrated the ability to elicit helper T-cell responses and to generate binding antibodies against all four proteins. Presently, it is undergoing animal model trials involving Rhesus macaques [247].

Among the DNA-based nucleic acid vaccines, the intradermal multivalent smallpox DNA vaccine, developed by Hirao et al., is notable for its utilisation of chemically synthesised and human codon-optimised VACV Western Reserve (WR) Strain genes, including A27, F9, H3, L1, A33, A56, and B5, in conjunction with the core antigen A4. This approach aims to enhance the vaccine’s cytotoxic T-cell activity [248].

In contrast, Mucker et al. have developed an mRNA-based vaccine utilising lipid nanoparticle delivery to administer unmodified mRNA encoding three monoclonal antibodies: c8A, c6C, and c7D11. This vaccine is undergoing preclinical animal studies to assess its immunogenicity and safety profile [249].

### 3.9. Preventive Measures of Mpox

The Mpox virus primarily spreads through direct contact with infected animals or person-to-person via respiratory droplets, bodily fluids, and skin lesions [61]. Understanding these transmission routes is essential for prevention, especially as the virus appears in non-endemic countries [7]. Effective prevention depends on rapid detection, which enables the isolation of infected individuals and reduces spread through symptom recognition and contact tracing [30,76]. Infected individuals should be isolated until they are no longer contagious, which is vital for outbreak control [250]. To reduce transmission and alleviate symptoms, consulting a healthcare provider, staying in well-ventilated areas, and practising good hygiene and sanitation are recommended.

Over-the-counter pain relievers such as paracetamol and ibuprofen can help relieve pain [251]. To prevent infections and the spread of viruses, popping blisters or scratching sores should be avoided, and maintaining isolation until all lesions have crusted over and scabs have fallen off is essential. Individuals should remain alert for symptoms for up to 21 days after exposure and take appropriate precautions [29]. Those with close contact with infected individuals should be quarantined to monitor for symptoms to prevent secondary transmission during outbreaks [30,252].

Healthcare professionals and individuals in close contact with infected persons should use PPE, including masks, gloves, and gowns [76,168,253]. It is essential to strictly adhere to disinfection protocols to prevent the spread of these infections. Orthopoxviruses can be inactivated by agents such as 70% ethanol (≤1 min), 0.2% peracetic acid (≤10 min), 1–10% probiotic cleanser (1 h), sodium hypochlorite (0.25–2.5%; 1 min), 2% glutaraldehyde (10 min), and 0.55% ortho-phthalaldehyde (5 min) [254].

Public health agencies must strengthen surveillance in both endemic and non-endemic areas [7,255], along with adequate environmental controls, such as educating communities to avoid contact with wild animals and to practice sanitation, to help reduce zoonotic Mpox transmission in endemic regions. Governments should issue travel advisories and provide guidance on preventive measures, such as avoiding contact with animals and maintaining good hygiene. Travellers must receive guidance on preventive measures, including avoiding contact with animals and maintaining strict hygiene practices [1,81]. Promoting behavioural changes, such as avoiding contact with suspected cases and practising proper hygiene and sanitation, is also essential [256,257]. Public awareness is essential; health campaigns should educate about symptoms, transmission, and prevention, increasing awareness and encouraging early healthcare visits [81,258,259].

In endemic areas, contact with animals such as primates and rodents that may carry Mpox should be strictly avoided to reduce the risk [260]. Educating the general public raises awareness and encourages safe hunting and handling [57]. Collaborating with veterinary services can help track and control Mpox in animals. Regular health checks and population monitoring are key to identifying potential sources of the virus [258]. Community leaders and healthcare professionals should work together to dispel misinformation and provide clear guidance on protective measures [259].

Limited public awareness of Mpox worsens due to social media misinformation, leading to fear, panic, and discrimination against key populations. This phenomenon, known as an ‘infodemic’, hinders response efforts by obscuring facts and marginalising experts, much as in the early stages of the COVID-19 pandemic [261,262,263,264]. A strategic approach involving policymakers, healthcare providers, the media, and social platforms is essential for raising awareness and reducing stigma. Public figures and medical professionals play crucial roles in reassuring the public and promoting health guidelines. Addressing research gaps and improving healthcare communication can build trust and encourage timely care. Emphasising a One Health approach is vital to combat stigma and strengthen public health efforts [261].

Effectively controlling Mpox mandates a multifaceted approach that includes public health education, vaccination, surveillance, and community involvement [7]. The successful implementation of these measures will reduce the likelihood of Mpox outbreaks and safeguard public health [76]. Furthermore, continuous research and effective coordination among health sectors are crucial for adapting and updating strategies as new information becomes available [255,265].

## 4. Future Perspectives and Recommendations

The ongoing global spread of Mpox, particularly highlighted by the 2022 outbreak, underscores significant deficiencies in diagnosis, treatment, and prevention strategies, as well as in healthcare infrastructure and facilities. These deficiencies pose formidable challenges for healthcare organisations and researchers alike. The absence of Mpox-specific antiviral drugs exacerbates these challenges, underscoring the urgent need for randomised controlled trials of promising antiviral candidates, including brincidofovir, tecovirimat, and novel agents such as ST-357. Special attention must be directed towards high-risk populations, including immunocompromised individuals, pregnant women, and communities in endemic regions, particularly in Africa. Moreover, vaccine development must extend beyond repurposing existing smallpox vaccines to encompass robust research initiatives and clinical trials focused on Mpox-specific vaccine candidates. This includes, but is not limited to, advancements in virus-like particles, mRNA vaccine technologies, and multi-epitope subunit vaccines targeting key antigens such as A29L, A35R, and L1R. Furthermore, ensuring equitable access to antiviral medications and vaccines globally is critical to mitigating the Mpox crisis.

The acceleration of research to develop more precise and rapid diagnostic approaches is paramount, particularly given the current reliance on clinical manifestations. While PCR remains the gold standard, its limited availability in resource-constrained settings underscores the need for rapid point-of-care testing platforms, including loop-mediated isothermal amplification (LAMP), CRISPR-Cas12 assays, and lateral flow tests targeting specific viral antigens. Strengthening real-time whole-genome sequencing capacities, particularly in endemic regions, is crucial for ongoing surveillance of viral lineages, tracking APOBEC3 mutations, and monitoring potential antiviral resistance.

Additionally, there is an urgent need to enhance public awareness and understanding of Mpox transmission, treatment, and prevention strategies. This is especially vital for populations in low- and middle-income countries, where vaccination rates may be suboptimal and reliance on personal protective measures remains critical. Healthcare professionals must receive improved training focused on both Mpox diagnosis and management, supported by community-oriented, non-stigmatising communication strategies to mitigate misinformation within high-risk groups. Further empirical research, including statistical and meta-analytic analyses in Mpox hotspots, is essential for systematically evaluating existing control strategies, therapeutic interventions, and vaccine efficacy.

Lastly, the establishment of effective global coordination, along with prioritisation of pre-positioned vaccines and antiviral supplies for endemic countries, is imperative. This should include incorporating Mpox-specific guidelines into national pandemic preparedness frameworks and investing in long-term healthcare infrastructure based on a One Health approach that integrates human, animal, and environmental health surveillance systems. Such comprehensive strategies are crucial for reducing transmission and preventing future outbreaks.

## 5. Limitations of This Review

As this article was intended to be a narrative review, a systematic review approach was not employed to select appropriate studies as data sources. This approach may have led to the omission of information that could have been relevant to the review. Additionally, the absence of quantitative data and statistical analysis may have limited the conclusions that could be drawn from the review.

## 6. Conclusions

Mpox, caused by the zoonotic Mpox virus, has been reported worldwide, particularly in West and Central Africa, since its discovery in 1958. The virus gained global attention during the worldwide outbreak in 2022, which resulted in millions of clinically diagnosed cases and hundreds of deaths. Mpox spreads through close contact with infected animals or humans, and after infection, the virus travels through the lymphatic system, causing symptoms that range from mild skin lesions to severe, potentially fatal complications affecting multiple organ systems. Diagnosis involves several laboratory techniques, including polymerase chain reaction (PCR), enzyme-linked immunosorbent assay (ELISA), immunohistochemical methods, and rapid detection techniques utilising biosensor devices. Treatment primarily consists of supportive care along with antiviral agents such as cidofovir, brincidofovir, and tecovirimat. For prevention, several vaccines originally developed for smallpox, such as Dryvax, LC16m8, and IMVAMUNE (also known as JYNNEOS or IMVANEX), are currently in use. In addition, preventive strategies include routine surveillance, increased public awareness, accurate diagnosis, strengthening healthcare systems, and implementing updated guidelines for the control, treatment, and prevention of Mpox, all of which are essential to limit the spread and progression of the disease.

## Figures and Tables

**Table 1:** Signs and symptoms of Mpox in different phases.

Major Phases	Periods	Symptoms	Sites of Lesions	Time Periods	References
Invasive	Incubation	Fever, chills, swollen lymph nodes, headache, backaches, muscle pain, exhaustion, crusting	Mouth, nose, pharynx, skin	Generally lasts 6–13 days, but can sometimes last up to 21 days.	[105,111]
	Prodromal	Fever, headache, itching, enlarged lymph nodes, myalgia, sore throat	Skin, mouth, nose	Lasts 1–4 days in most cases. Primary fever after the rash can last 2–3 days, and secondary fever can occur during the rash and last 2–3 days.	[16,17,80]
Cutaneous	Rash	Macules, papules, vesicles, pustules, crust and scabs	Face, arms, legs, genitals, scalp, palms, soles of feet, mouth, eyes.	Rashes usually appear 1–3 days after fever onset. Each stage lasts 1–2 days, while the pustular phase may last 5–7 days. Macules can persist for 2–4 weeks.	[49,63,105]
	Crusting	Erythema or pigmentation, paraphimosis, necrotic crust, proctitis	Oral mucosa, pharyngeal wall, tongue, tonsils	Scab shedding usually occurs within 2–4 weeks after the initial onset, but can extend up to 8 weeks in some cases.	[63,112]

**Table 2:** Laboratory test methods available for the precise diagnosis of Mpox.

Test	Working Principle	Sample Type	Detection Time	Advantages & Limitations	References
Electron microscopy	Morphologically identifies the Mpox virus	Skin specimens/lesion	Around 1 week	Accurate, expensive	[140,141,142]
Virus isolation, culture and CPE screening	Uses HeLa, Vero, BSC-1, and RK-13 cell lines and chicken embryos to grow the Mpox virus and detect it through CPE screening methods	Lesion	Around 1–4 days, sometimes more	Expensive, trained staff and BSL3 level lab required, time-consuming	[129,141,142]
PCR	Detects and verifies the existence of Mpox DNA in the sample.	Skin specimen/lesion	3–5 h	Sensitive, rapid, inexpensive	[17,130,143]
Real-time PCR	Detects Mpox-specific conserved portions of the extracellular envelope protein gene (*B6R*), DNA polymerase gene, and *E9L* gene	Virus isolates	1–3 h	Highly sensitive, specific, rapid, inexpensive	[134,138,139]
ELISA	Detects Mpox by detecting particular antibodies in Mpox-infected patients’ serum	Serum	3–4 h	Incapable of type differentiation	[62,109]
CRISPR	Detects viral nucleic acid through a CRISPR-Cas12-based reverse-transcriptase- mediated isothermal amplification approach	Virus isolates	Around 35 min	Technological research in progress	[144,145,146]
LAMP	Amplifies DNA using *Bst* DNA polymerase and specific primer sets under isothermal conditions	Lesions, crusts, swabs	30–60 min	Efficient, specific, no need for thermal cycling equipment, complex primer designing	[113,147,148,149]
RPA	Works through isothermal DNA amplification based on the activities of recombinases, single-stranded binding proteins and polymerases	Lesions, crusts, swabs	5–15 min	No need for a complex thermocycler, conducts reaction at low temperature for a short time	[149,150,151]
HIA	Detects viral antigens through virus-specific antibodies	Serum	30–60 min	Simple, inexpensive, needs fresh red blood cells, not sufficiently specific	[149,152]
WB	Separates viral DNA through agarose gel electrophoresis	Virus isolates	3–4 h	High amount of sample required, limited sample throughput	[130,135,143]
Whole genome sequencing	Detects Mpox based on metagenomic sequencing or tiled-PCR amplification	Virus isolates	5–10 days	Accurate, expensive, time-consuming	[132,136,153]
Cepheid GeneXpert system	An analytical workstation that can detect the Mpox virus through combined sample preparation and real-time PCR amplification	Virus isolates	About 1 h	Rapid POC testing, reduced contamination, and minimised sample amount	[143,154]

Abbreviations: PCR—polymerase chain reaction; CRISPR—clustered regularly interspaced short palindromic repeats; LAMP—loop-mediated isothermal amplification; RPA—recombinase polymerase amplification; HIA—hemagglutination inhibition assay; WB—Western blot; ELISA—enzyme-linked immunosorbent assay; CPE—cytopathic effect; BSL—biosafety level; POC—point-of-care; HeLa: Henrietta Lacks; Vero: verda reno; BSC-1: Biologics Standards-Cercopithecus-1; RK-13: Rabbit Kidney-13.

**Table 3:** Major antiviral drugs to treat Mpox.

Antiviral Drug	Trade Name	Trade Number	First Manufacturing Company	Mode of Action	Administration Route	Formulations and Doses	Animal Models	Trial on Human	FDA Approval Status	References
Tecovirimat	TPOXX	NDA 208627	SIGA Technologies (New York, NY, USA)	Inhibits VP37 envelope wrapping protein, prevents virus maturation, and release	Oral, intravenous	600 mg, three capsules orally, twice a day for 14 days	Monkeys	Yes	Smallpox (2018)	[172,179,186]
Brincidofovir	Tembexa	NDA 214460	Chimerix (Durham, NC, USA)	Inhibits viral DNA polymerase interrupts viral replication.	Oral	10 mg/mL oral suspension, 100 mg tablets	Rabbits, prairie dogs	Yes	Smallpox (2021)	[175,176,186]
Cidofovir	Vistide	NDA 020638	Gilead Sciences (Foster City, CA, USA)	Inhibits viral DNA polymerase, prevents viral replication	Intravenous	5 mg/kg intravenously once a week for two weeks, a 375 mg/5 mL vial for injection.	Mice	Yes	CMV (1996)	[173,174,186]

Abbreviations: NDA—new drug application; mg—milligram; mL—milliliter; FDA—food and drug administration; CMV—cytomegalovirus.

**Table 4:** Major vaccines of different generations are used to prevent Mpox.

Vaccine	First Manufacturing Company	Generation	Vaccine Type	Type of Virus Used	Mode of Action	Number of Doses	Interval Between Doses (Days)	Animal Models	Trial on Human	FDA Approval Status	Ref
Dryvax	Wyeth Laboratories, Inc. (Marietta, PA, USA)	1st generation	Live vaccinia virus-based vaccine	Replication-competent vaccinia virus strain	Boosts humoral and cell-mediated immune responses. Induces neutralising antibody and T cell responses.	1	Single dose (No interval)	Mice, Rabbits and Monkeys	Yes	Smallpox (1931)	[213,214,215]
Aventis Pasteur Smallpox Vaccine (APSV)	Sanofi-Aventis (Bridgewater, NJ, USA)	2nd generation	Live vaccinia virus-based vaccine	Replication-competent vaccinia virus strain	Boosts humoral and cell-mediated immune responses. Induces neutralising antibody and T cell responses.	1	Single dose (No interval)	NR	Yes	Authorised as IND/EUA	[217,218,219]
ACAM2000	Acambis, Inc (Cambridge, UK and Cambridge, MA, USA)	2nd generation	Attenuated vaccinia virus-based vaccine	Replication-competent vaccinia virus strain	Boosts humoral and cell-mediated immune responses. Induces neutralising antibody and T cell responses.	1	Single dose (No interval)	Mice, prairie dogs, rabbits and monkeys	Yes	Smallpox (2007)	[220,221,222]
LC16m8	KM Biologics Co., Ltd. (Kumamoto, Japan)	3rd generation	Attenuated vaccinia virus-based vaccine	Replication-competent Lister strain of vaccinia virus	Boosts humoral and cell-mediated immune responses. Induces neutralising antibody and T cell responses.	1	Single dose (No interval)	Mice, Rabbits and Monkeys	Yes	No	[223,224,225,226]
JYNNEOS	Bavarian Nordic A/S (Hellerup, Denmark)	3rd generation	Attenuated vaccinia virus-based vaccine	Replication-deficient MVA strain	Boosts humoral and cell-mediated immune responses. Induces neutralising antibody and T cell responses.	2	28	Mice, Rabbits and Monkeys	Yes	Smallpox and Mpox (2019)	[222,227,228,229]

Abbreviations: MVA—modified vaccinia ankara; EUA—emergency use authorisation; IND—investigational new drug; FDA—food and drug administration; NR—not reported.

## Data Availability

This article is a narrative review based exclusively on previously published literature. No new datasets were generated or analyzed during this study; therefore, data sharing is not applicable.
